# How to explain the beneficial effects of leukocyte‐ and platelet‐rich fibrin

**DOI:** 10.1111/prd.12570

**Published:** 2024-06-24

**Authors:** Juan Blanco, Angel García, Lidia Hermida‐Nogueira, Ana B. Castro

**Affiliations:** ^1^ Department of Surgery (Stomatology, Unit of Periodontology) Universidade de Santiago de Compostela Santiago de Compostela Spain; ^2^ Platelet Proteomics Group, Center for Research in Molecular Medicine and Chronic Diseases (CIMUS) Santiago de Compostela University Santiago de Compostela Spain; ^3^ Department of Oral Health Sciences, Section of Periodontology, KU Leuven & Dentistry University Hospitals Leuven Leuven Belgium

**Keywords:** analgesic, antibacterial, antimicrobial, bone healing, bone regeneration, hemostatic, leukocyte‐ and platelet‐rich fibrin, pain, soft tissue regeneration, wound healing

## Abstract

The survival of an organism relies on its ability to repair the damage caused by trauma, toxic agents, and inflammation. This process involving cell proliferation and differentiation is driven by several growth factors and is critically dependent on the organization of the extracellular matrix. Since autologous platelet concentrates (APCs) are fibrin matrices in which cells, growth factors, and cytokines are trapped and delivered over time, they are able to influence that response at different levels. The present review thoroughly describes the molecular components present in one of these APCs, leukocyte‐ and platelet‐rich fibrin (L‐PRF), and summarizes the level of evidence regarding the influence of L‐PRF on anti‐inflammatory reactions, analgesia, hemostasis, antimicrobial capacity, and its biological mechanisms on bone/soft tissue regeneration.

## 1 INTRODUCTION

**FIGURE 1:**
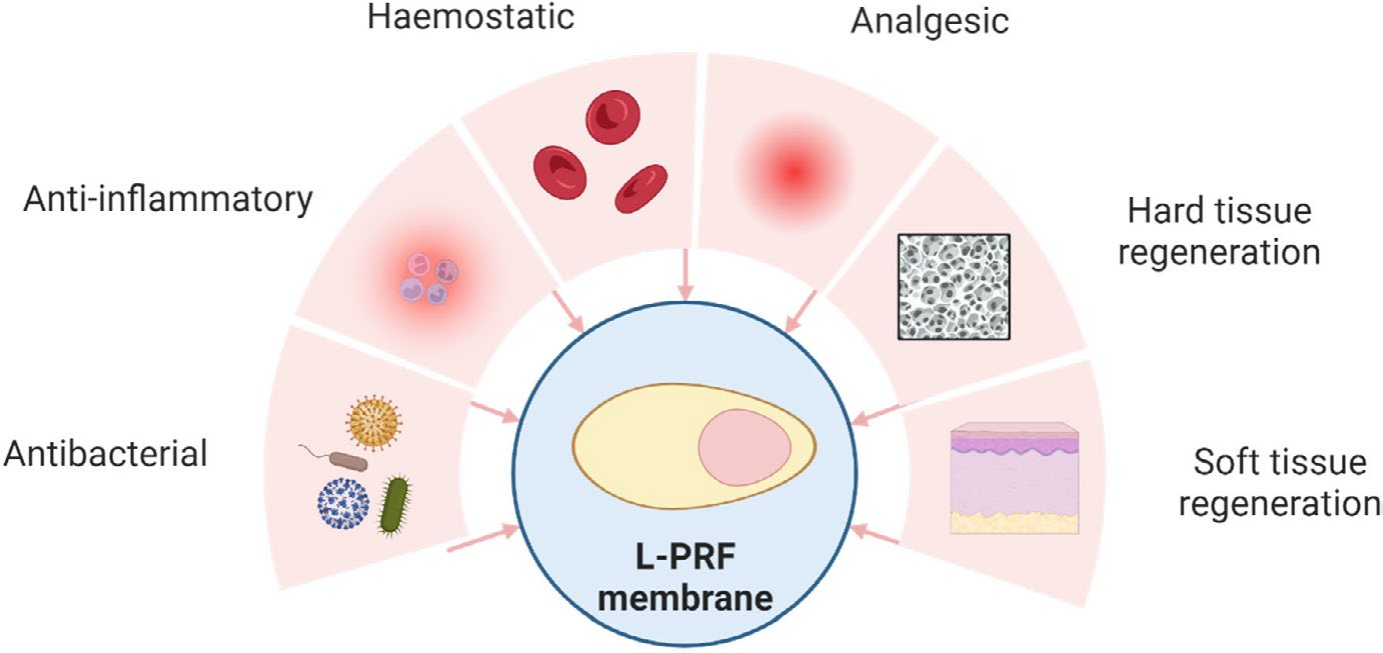
Schematic illustration of the effects of leukocyte and platelet rich fibrin.

Human blood represents about 8% of the total body weight. It is formed in the blood marrow and consists of two basic components: plasma (about 55%) and formed elements (about 45%).^1^ Plasma is the liquid portion of blood and about 90% is water. It contains many dissolved substances and three types of formed elements: white blood cells (1%), red blood cells (40%–45%), and platelets (<1%).^2^ During natural wound healing, the unfractionated blood clot (UBC) fills the defect site. Leukocyte‐ and platelet‐rich fibrin (L‐PRF) is obtained through centrifugation of a blood sample and as a result, platelets and white blood cells remain at high concentration in the L‐PRF clot. These cells will be the source of different molecules that are released and entrapped in the fibrin matrix which will enhance different processes described below (Figure 1). However, it is important to mention that although both UBC and L‐PRF have potent TGF‐β and anti‐inflammatory activity, UBC does not have the strength properties required to be used clinically to prepare applicable membranes. Thus, centrifugation (eliminating the erythrocytes) is necessary to generate durable and clinically applicable blood‐derived membranes.^3^, ^4^

## 2 PLATELETS

Platelets are derived from the cytoplasmic fragmentation of megakaryocytes. They are enucleated cell fragments 1–3 μm in diameter that serve as a reservoir for a wide variety of molecules, and their survival ranges from 7 to 10 days. Following damage to vascular or tissue integrity, circulating platelets are the first cellular response and play a crucial role in hemostasis, innate immunity, angiogenesis, and wound healing. The latter aspect is receiving increased attention as the wound‐healing effects suggest a regenerative ability for maintaining whole‐body integrity and homeostasis.^3^

Platelet activation can be initiated by a variety of physical (platelet adhesion with high shear forces) or chemical stimuli. Platelet agonists are diverse and include nucleotides such as adenosine diphosphate (ADP), amines such as epinephrine and serotonin, lipids such as prostaglandins, thromboxanes, and platelet‐activating factor, as well as proteins such as collagen and thrombin. The latter is the most potent platelet agonist.^5^

In addition to minimizing blood loss, once activated, the platelets promote healing of the damaged tissue. This is achieved through the release of cytokines, chemokines, and growth factors from platelet granules. There are three major types of secretory granules in platelets including the following: (i) alfa‐granules, containing many growth factors and cytokines; (ii) dense granules, which release calcium, serotonin, polyphosphates, pyrophosphates, adenosine diphosphate (ADP), and adenosine triphosphate; and (iii) lysosomes, which contain a number of hydrolytic enzymes. There are approximately 50–80 alfa‐granules per platelet with a typical diameter of 200–500 nm that can be released intracellularly or extracellularly. Growth factors found in the platelet releasate are involved in promoting tissue regeneration, such as endothelial growth factor (EGF), which causes cell growth, recruitment, and differentiation as well as cytokine exocytosis and secretion. Similarly, the growth factor platelet‐derived growth factor‐BB (PDGF‐BB) (homodimers PDGF‐AA, PDGF‐BB, PDGF‐CC, and PDGF‐DD and the heterodimer PDGF‐AB) has a physiological effect that causes significant cell growth, cell migration, blood vessel growth, granulation, growth factor secretion, and matrix formation with bone morphogenetic proteins (BMPs).^3^ However, some discrepancies in the releasate content may be accounted for by the varying methods used for activating platelets. In this context, four main types of platelet activation (i.e., 10% of collagen type I, calcium chloride [CaCl_2_], autologous thrombin, or a mixture of CaCl_2_ and thrombin) may have an impact on the amount of growth factors and cytokines (e.g., transforming growth factor‐β1 [TGF‐β1], tumor necrosis factor‐α [TNF‐α], interleukin‐1beta [IL‐1β], PDGF‐AB, and vascular endothelial growth factor [VEGF]) released by activated platelets, when collecting releasates specifically for regenerative applications.^6^ Standardization of platelet activation is a crucial step to optimize the various growth factors and cytokines released and implemented in experimental procedures. Different protocols for different tissue or cell types have been outlined. In fact, only the first‐generation APCs require the use of an activator in comparison with the physiological coagulation process activation of the second‐generation APCs. Other experimental variables such as platelet count, centrifugation speed, and type of collection tube have also been recognized as having an impact on the platelet releasate.^7^, ^8^

## 3 LEUKOCYTES

Healing is an interactive process that involves soluble mediators, extracellular matrix components, resident cells including platelets, and infiltrating leukocyte subtypes, which participate in different manners in the four phases of wound healing: coagulum, inflammation, tissue formation, and tissue remodeling.^9^

After the neutrophils' extravasation at the injury site, they migrate to the wound and are captured in the fibrin net together with additional actively recruited neutrophils from adjacent blood vessels to form a dense barrier against invading pathogens and counteract flare‐up infection (antimicrobial effect).^10^ Their main role is to produce inflammatory cytokines and a battery of growth factors. At day 1 after tissue injury, nearly 50% of all cells at the wound site are neutrophils.^11^ The studies suggest that the peak neutrophil infiltration in the damaged soft tissue occurs within 24 h postinjury and is associated with both maximum fiber tearing and oxidant production.^12^, ^13^ Monocytes/macrophages invade the wound area concomitantly and they play a central role in wound repair.^14^, ^15^ They exhibit immunological functions as antigen‐presenting cells, phagocytes, and are an important source of growth factors.^9^, ^16^ Macrophages also secrete collagenase, which stimulates the process of cleaning the wound, and excrete transforming growth factors (TGF) to stimulate the keratinocytes, as well as platelet‐derived growth factors (PDGF). They also release IL‐1, fibroblast growth factor (FGF), and tumor necrosis factor (TNF), which are substances that stimulate fibroblasts to produce collagen and improve angiogenesis.^17^ Lymphocytes also produce growth factors, and they may contribute to tissue remodeling during the late phase of wound healing.^18^, ^19^ Therefore, the sources of essential healing molecules come not only from platelets but also from leukocytes.

In the L‐PRF biomaterial, the effects of leukocytes and the fibrin matrix are deeply interconnected.^20^ The L‐PRF clot and membrane contain at least 50% of the leukocytes from the initial blood harvest, and these cells are enmeshed in the fibrin matrix following a specific three‐dimensional cell distribution.^21^ The lymphocytes are more concentrated than the other leukocytes. The overpopulation of lymphocytes in this technique is of particular interest^20^ because lymphocytes are turntables of the local regulation during healing, and this may explain why the L‐PRF membrane can continue to produce large quantities of growth factors during a long period. An L‐PRF membrane releases significant amounts of growth factors and matrix proteins during more than 7 days,^22^, ^23^, ^24^, ^25^, ^26^ and leukocytes seem to be the source of the overproduction of some of these growth factors (particularly VEGF and transforming growth factors^22^).

On the other hand, there is controversy on the role that leukocytes play in these autologous platelet concentrates. There are some groups that suggest the possible negative effects of using platelet‐rich plasma with leukocytes in tissue regeneration because a high concentration of proinflammatory cytokines would increase the adverse effects of the inflammatory process, such as pain and swelling.^27^, ^28^ Moreover, leukocytes contain and produce biologically active cytokines that are predominantly catabolic or inflammatory, reactive oxygen species (ROS), and proteases, including collagen‐degrading matrix metalloproteinases.^29^, ^30^, ^31^, ^32^ However, in recent clinical studies and systematic reviews on the efficacy of different types of platelet‐rich plasma (leukocyte poor vs. leukocyte rich) in the management of lateral epicondylitis^33^, ^34^, ^35^ and the treatment of knee osteoarthritis,^36^, ^37^, ^38^ the results showed either no difference between the two groups or in favor of the leukocyte‐rich PRP in terms of pain and functional scores. Moreover, recent in vitro studies showed that liquid PRF, solid L‐PRF membranes, lysates, and L‐PRF conditioned medium can hold an anti‐inflammatory activity, shift the macrophage polarization from macrophage‐1 (M1) toward an M2 phenotype, and inhibit the formation of osteoclasts (osteoclastogenesis) from hematopoietic progenitors in murine bone marrow cultures.^39^, ^40^, ^41^

Another important role of the leukocytes in the APCs is its antimicrobial effect. L‐PRF showed bacterial growth inhibition against the main periodontal pathogens (*Aggregatibacter actinomycetemcomitans*, *Porphyromonas gingivalis*, *Prevotella intermedia*, and *Fusobacterium nucleatum*).^42^, ^43^, ^44^, ^45^ However, preparation protocols differed enormously among studies and may hinder the real comparison between them.

## 4 FIBRIN NETWORK

Fibrinogen is a soluble fibrous glycoprotein normally present in human blood plasma at a concentration of about 2.5 g/L and is essential for hemostasis, wound‐healing, inflammation, angiogenesis, and other biological functions.^46^ The liver is the primary source of plasma fibrinogen, with a steady‐state rate of synthesis of 1.7–5.0 g per day and a large reserve.^47^ Three‐quarters of the human fibrinogen is located in the plasma, but it is also present in platelets, lymph nodes, and interstitial fluid. The half‐life of fibrinogen is 3–5 days.^46^

**FIGURE 2:**
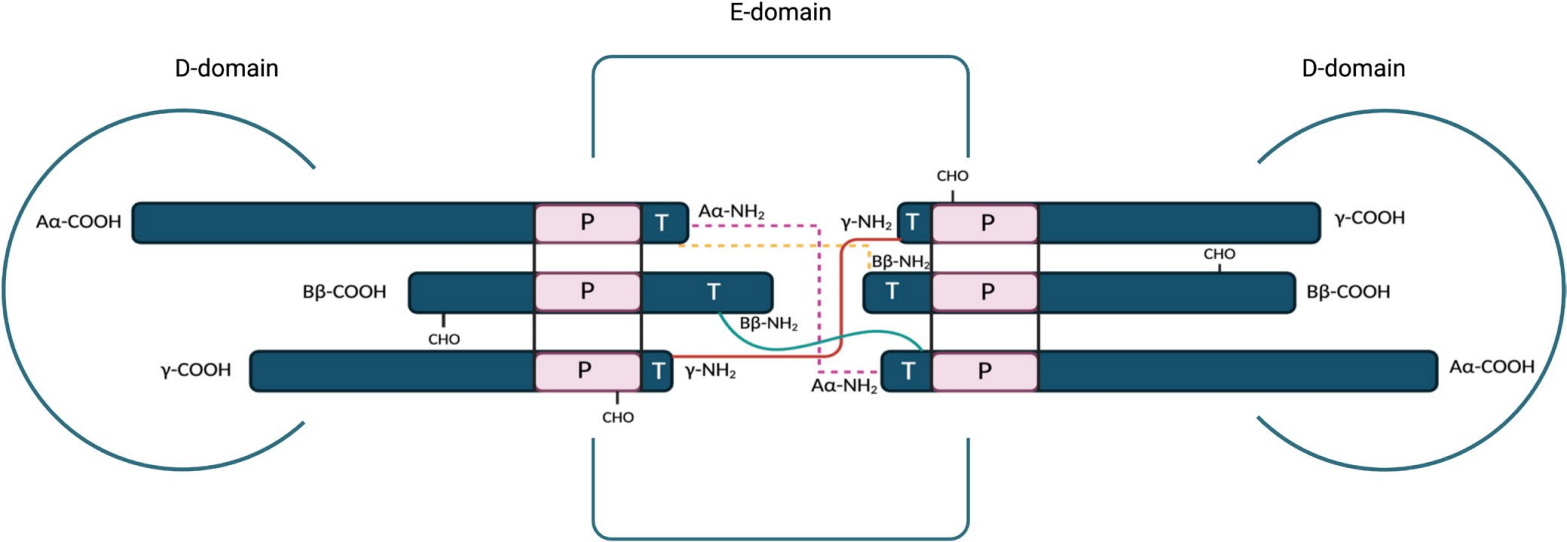
Schematic diagram of the three pairs of polypeptide chains (Aα, Bβ, and ϒ) of fibrinogen. Indicated in the figure the location for carbohydrate attachment (CHO), thrombin (T) and plasmin cleavage (P). Aα, Bβ and γ chains form the D‐domain and these are joined by α‐helical ropes to the central E‐domain to give the characteristic fibrinogen structure.

Fibrinogen molecules (Figure 2) are elongated 45 nm structures that consist of two outer D domains, each connected by a coiled‐coil segment to its central E domain. The fibrinogen molecule is comprised of two sets of three polypeptide chains termed Aα, Bβ, and ϒ, which are joined together in the N‐terminal E domain by five symmetrical disulfide bridges.^48^ The fibrinopeptides, which are in the central region (E domain), are cleaved by thrombin to convert soluble fibrinogen to an insoluble fibrin polymer, via intermolecular interactions of the “knobs” exposed by fibrinopeptide removal with “holes” always exposed at the ends of the molecules. Fibrin monomers polymerize via these specific and tightly controlled binding interactions to make half‐staggered oligomers that lengthen into protofibrils. The protofibrils aggregate laterally to make fibers, which then branch to yield a three‐dimensional network—the fibrin clot—essential for hemostasis. Finally, the transglutaminase, factor XIIIa, covalently binds specific glutamine residues in one fibrin molecule to lysine residues in another via isopeptide bonds, stabilizing the clot against mechanical, chemical, and proteolytic insults.^46^

The mechanical properties of fibrin are essential for its functions in hemostasis and wound healing since the clot must stop bleeding and yet allow the penetration of cells.^46^ These physical and mechanical characteristics of clot networks vary greatly depending on the conditions of the fibrin polymerization. Many factors can have a great impact on the rates of various steps in the polymerization process, such as thrombin concentration (affecting the rate of generation of fibrin monomer by cleavage of fibrinopeptides), pH, calcium, chloride ion concentration, and other plasma proteins.^46^

**FIGURE 3:**
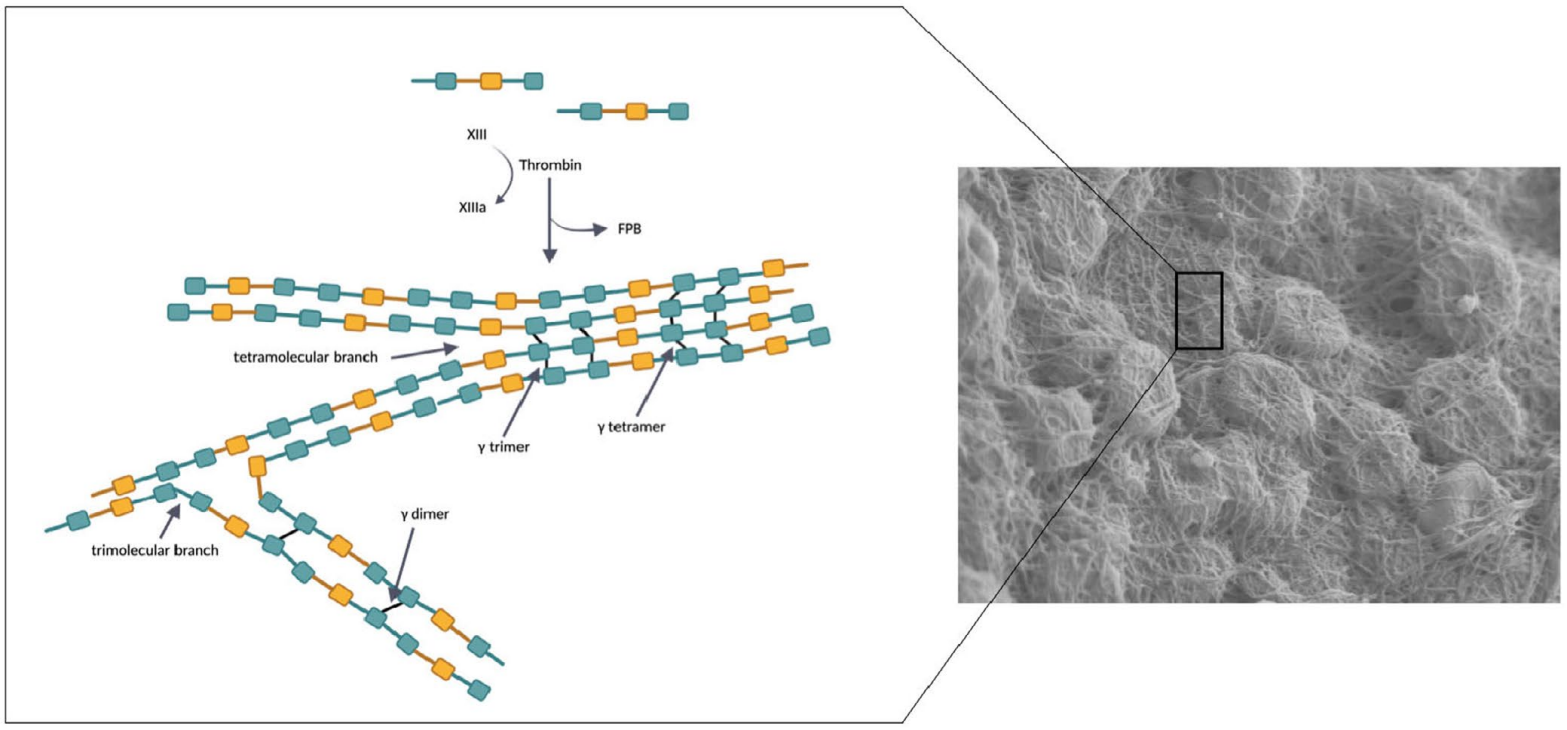
Diagram illustrates the process of fibrin assembly and crosslinking. Fibrils exhibit branching and engage in lateral associations to create broader fibrils and fibers. Factor XIII or its activated form, XIIIa, facilitates the formation of ε‐(γ‐glutamyl) lysine isopeptide bonds, leading to the swift development of γ‐dimers. The formation of γ‐trimers and γ‐tetramers occurs more gradually through interfibril γ‐chain crosslinking, enhancing the clot's resistance to fibrinolysis.

Once the protofibrils aggregate laterally to make fibers, they branch to obtain a complex fibrin network structure. Two types of branching account for fibrin network structures, and they both play important roles in defining clot network structure. The first type occurs when double‐stranded fibrils converge side to side to form the most widely appreciated fibril junction, a tetramolecular or bilateral branch point. More extensive lateral fibril associations result in large fiber bundles consisting of multiple fibrils and more condensed bilateral branch structures. This type of structure confers strength, rigidity, and denser‐network fibers. The second type of branch junction, termed trimolecular or equilateral, forms by the coalescence of three fibrin molecules that connect three double‐stranded fibrils of equal widths (Figure 3). Equilateral branches probably form with greater frequency when fibrinopeptide cleavage is relatively slow. The networks are more branched, less porous, form a tighter fibrin, and enhance clot elasticity. This kind of branch junction is obtained at low levels of thrombin and with a slow physiologic fibrin polymerization process.^49^, ^50^ This aspect can be very important in the characteristics of the fibrin which constitutes part of the L‐PRF products and should be taken into account when obtaining the final biomaterial after centrifugation. In fact, bilateral junctions are provoked by a drastic activation and polymerization, for example, with high thrombin concentrations, which leads to a dense network of monofibers similar to a fibrin glue, which is not particularly favorable to cytokine enmeshment and cellular migration. On the contrary, a slow physiological fibrin polymerization (such as in L‐PRF) yields a higher percentage of equilateral junctions, which allows the establishment of a flexible fibrin network with multifiber assembly that is capable of supporting cytokine enmeshment and cellular migration and acts also as an artificial stem cell niche containing hematopoietic and multipotent cells (as a reservoir), similarly to bone marrow and perivascular niches.^51^ Moreover, this organization will also provide elasticity to the fibrin matrix comparable to that of a solid biomaterial. Fibrinogen collection efficiency and polymerization type define the material characteristics of the concentrate.^52^

On the other hand, L‐PRF membranes typically have very short resorption times, ranging from a 7‐day^53^ to 28‐day^54^ period, and its ability to maintain space (stiffness) is compromised. This is why these membranes preferably should not be used alone as a barrier for bone regeneration. However, during their limited resorption time, a slow and gradual release of growth factors is observed within the L‐PRF matrix. During guided bone regeneration (GBR) procedures (most notably during extensive GBR cases), L‐PRF membranes should then be combined with either a collagen barrier membrane or titanium/titanium‐reinforced membranes. In this context, L‐PRF membranes can be placed over or under the barrier membrane. As L‐PRF is known to rapidly promote greater soft tissue wound healing, its biological use involves contact with soft tissues on the outer surface of barrier membranes. However, it would be advantageous to place the L‐PRF membrane under a nonresorbable membrane as the periosteum would not be able to supply angiogenesis through this membrane, and L‐PRF placed under this membrane can supply the early growth factors responsible for new blood vessel formation within the underlying bone augmentation procedure. Therefore, L‐PRF should be placed above or under the barrier membrane and in noncontained or extensive defects, and should not be used as a barrier membrane by itself.^55^

## 5 PROTEOME

Some important biological processes, such as cell migration/differentiation, angiogenesis, and extracellular matrix synthesis, are regulated by various growth factors, cytokines, and other proteins which predominantly originate from platelets and leukocytes in the fibrin clot during the wound‐healing process. In each phase of this process, different cell types at different times take part in order to regenerate the tissue.^56^, ^57^, ^58^

According to the current knowledge, L‐PRF comprises platelets, leukocytes, some circulating stem cells, growth factors, cytokines, other proteins, and a fibrin network that acts as a biological scaffold and a reservoir as it postoperatively enables sustained release of these molecules to an extent.^22^, ^59^ These are considered to contribute to the regenerative potential.^60^

Tissues in the body comprise various abundant proteins related to their extracellular matrix contents; this makes studying proteins at a tissue level more complicated than studying proteins in cultured cells.^61^ After protein extraction, all proteins in the investigated tissue sample remain in the same test tube. Therefore, due to the domination of abundant proteins in the investigated tissue samples, it is difficult to identify proteins that exist in very small amounts. The use of the right and modern proteomic methods of analysis to decipher the proteome profile of any tissue is of utmost relevance.

To date, some studies have quantified and measured the kinetics of specific growth factors released by platelets and leukocytes in the L‐PRF membranes^62^, ^63^, ^64^; however, cells release many other proteins, which contribute to regeneration. In fact, and due to the limited number of studies aiming to analyze the proteome of L‐PRF, we have considered and compared all types of APCs (excluding platelet concentrates used in transfusions). These are described chronologically due to the natural development of the proteome methods of analysis applied^65^, ^66^, ^67^, ^68^, ^69^ (Table 1).

**TABLE 1:** Most relevant studies describing proteomics in PCs.

Study (year)	Platelet concentrated preparation	Method	Timing of analysis	Main outcome
Anitua et al. (2015)^65^	PRGF protocol 580 g/8 min + sodium citrate + CaCl_2_ F1 (poor in platelets), F2 (discarded), and F3 (rich)	Double approach (1D SDS‐PAGE + LC‐MS/MS protein identification/2‐DE + MALDI‐TOF/TOF protein identification)	Cross‐sectional	Proteins (1D SDS‐PAGE + LC‐MS/MS, 136 in F1 and 142 in F3 proteins and with 2‐DE + MALDI‐TOF/TOF, 49 in F1 proteins) identification pathways (acute‐phase response signaling and lipid metabolism)
Yaprak et al. (2018)^66^	L‐PRF 400 g/10 min	2‐DE + MALDI‐TOF/TOF MS/MS	Cross‐sectional	35 proteins (complement proteins, serine protease inhibitors, immunoglobulins, and some acute‐phase proteins)
Lee et al. (2020)^67^	Leukocyte poor PRP^a^ 380 g/5 min – Leukocyte‐rich PRP^b^ 750 g/15 min –	1D SDS‐PAGE + LC‐MS/MS analysis	Cross‐sectional	664 proteins (125 related to wound healing) Of the 664 proteins, 379 were identified in the LP‐PRP and 618 in the LR‐PRP
Di Suma et al. (2020)^69^	PRF 400 g/12 min	LC‐MS/MS analysis	Cross‐sectional	650 proteins identified in PRF lysates; only few growth factors reported, being TGF‐β the most relevant one
Hermida‐Nogueira et al. (2020)^68^	L‐PRF 400 g/12 min Intraspin centrifuge	Qualitative analysis of secretomes at days 3 and 7 (1D SDS‐PAGE + LC‐MS/MS) and quantitative analysis at 3, 7, and 21 days (Sequential Window Acquisition of all Theoretical mass spectra—SWATH)	3, 7, and 21 days	705 proteins secreted at day 3: growth factors (EGF and PDGF‐A) and proteins related to platelet and neutrophil degranulation 202 proteins quantified by SWATH: MMP9, TSP1, and CO_3_ at day 3 Fibrinogen and CATS down‐regulated at day 3 Identificated pathways: clathrin‐mediated endocytosis, acute‐phase response signaling, and LXR/RXR activation

Abbreviations: EGF, endothelial growth factor; mmp9, matrix metallopeptidase‐9; PDGF‐A, platelet‐derived growth factor‐a; TSP1, thrombospondin‐1; CO_3_: carbon trioxide.

^a^ Autologous conditioned plasma (ACP) double syringe system.

^b^ Gravitational platelet separation system (gps).

Anitua et al.^65^ described (cross‐sectional analysis) the proteomic characterization of plasma‐rich growth factors (PRGF). From three healthy volunteers, venous blood was withdrawn. The whole‐blood samples were processed according to the PRGF‐Endoret protocol.^70^ After protein extraction, samples (fractions 1 and 3, poor and rich in platelets, respectively) were processed and analyzed following a dual qualitative approach (1D SDS‐PAGE + LC‐MS/MS/2‐DE + MALDI‐TOF/TOF). The LC‐MS/MS technique identified 136 proteins in fraction 1 and 142 in fraction 3, with an overlap of 111 proteins. On the other hand, with the MALDI‐TOF/TOF approach, 49 unique proteins, present in 136 gel spots, were identified in fraction 1. As expected, this latter approach led to less proteins identified than the LC‐MS/MS one. The most represented canonical pathways related to all the proteins identified in fraction 1 were mainly related to the restoration of homeostasis, tissue repair, and wound healing, and included acute‐phase response signaling, complement system, and coagulation cascade.

Yaprak et al.,^66^ again in a cross‐sectional analysis, described the proteomic characteristics of the L‐PRF obtained from venous blood from eight healthy volunteers and after a 10‐min centrifugation at 400 g. The method for the proteomic analysis was based on 2‐DE + MALDI‐TOF/TOF and the main results were the identification of 35 unique proteins, distributed in 55 gel spots, including complement proteins, serine protease inhibitors, immunoglobulins, and some acute‐phase response proteins. Sixteen of the proteins identified have been previously reported to be linked with the wound‐healing process.

Three studies approaching the analysis of PRP and PRF proteomes and L‐PRF secretomes were published in 2020. Lee et al.^67^ analyzed the proteome (1D SDS‐PAGE + LC‐MS/MS) of two types of platelet‐rich plasma (PRP), poor and rich in leukocytes. The obtained results reflect more quantity of proteins than in the previous studies, probably due to the method of analysis and the inclusion of leukocytes. Overall, a total number of 664 proteins were consistently identified; 125 of which related to the wound‐healing processes, such as angiogenesis, fibroblast migration, collagen biosynthesis, and glycosaminoglycan biosynthesis.

Focusing on platelet‐rich fibrin (PRF) membranes, Di Summa et al.^69^ analyzed the proteome (LC‐MS/MS) of PRF lysates (PRF, 12 min at 400 g), and identified 650 proteins, being TGF‐β one of the few growth factors found. Interestingly, an RNAseq of gingival fibroblasts exposed to PRF lysates revealed 51 genes that were differentially expressed including IL‐11, NOX4, and PRG4, which are classical TGF‐β target genes.

Finally, Hermida‐Nogueira et al.^68^ deciphered the whole collected secretome of leukocyte‐ and platelet‐rich fibrin membranes (L‐PRF 12 min at 400 g) at days 3, 7, and 21 of culture (in DMEM).^71^ The method used was double: (1) qualitative analysis at days 3 and 7 (1D SDS‐PAGE + LC‐MS/MS) plus a quantitative growth factor analysis (Quantibody Human Growth Factor Array with 40 growth factors) to complement and corroborate the qualitative proteomic data, and (2) quantitative analysis at days 3, 7, and 21 (by Sequential Window Acquisition of all Theoretical mass spectra—SWATH). The main results indicated that at day 3 of culture, 705 proteins were identified, including growth factors (EGF and PDGFA) and proteins related to platelet and neutrophil degranulation as expected during the first days of the wound‐healing process. When the proteomic analyses compared the secretome between days 3 and 7, in the most differentiated four bands after 1D SDS‐PAGE, a total of 371 proteins were found at day 3 and 292 at day 7, from which 259 in both conditions. Common identifications belong to secretory pathways; indeed, proteins such as CD9, ITA2B, CAP7, and CATG are related to platelet‐ and neutrophil‐derived extracellular vesicles. Growth factors such as EGF and EGF‐containing fibulin‐like extracellular matrix protein 1 (FBLN3) were only identified at day 3. On the contrary, leukocyte adhesion proteins (intercellular adhesion molecule 3 [ICAM3] and myosin light polypeptide 6 [MYL6]) were only found on day 7. According to this analysis, most growth factors were found enriched at day 3, most probably due to a higher platelet and neutrophil degranulation at this time point, and the proteins found at day 7 are in relation with the evolution of the wound healing to next phases. At day 7, the number of macrophages probably is higher than at day 3, for that reason, the amount of these proteins is increased at that day. In the quantitative analysis, a total of 202 differentially secreted proteins were quantified by SWATH when comparing secretomes at days 3, 7, and 21. Most of them were enriched at day 3 such as MMP9, thrombospondin‐1 (TSP1), and CO3. On the contrary, fibrinogen and CATS were found to be down‐regulated on day 3. The most relevant pathways related to the proteins identified (systems biology analysis) were clathrin‐mediated endocytosis, acute‐phase response signaling, and LXR/RXR activation.

In the five studies here reviewed, there are important differences that make comparison between them, at least, difficult. On one hand, the study performed by Yaprak et al.^66^ found a low number of identifications, only 35, in L‐PRF releasates, whereas the other four studies,^65^, ^67^, ^68^, ^69^ provided a much more comprehensive analysis of platelets‐rich concentrates. However, the different protocols followed for sample generation and proteomic method of analysis make it difficult to extract a conclusion. Since the study by Hermida‐Nogueira et al.^68^, ^71^ is the most complete (whole secretome), and done at different time points (3, 7, and 21 days), it could be considered the most relevant. In fact, the authors conclude that the secretome profile at day 3 and the growth factors analysis (ELISA array) performed on days 3 and 7 showed that EGF, PDGFA, TGFB1, and proteins related to platelet and neutrophil degranulation might be responsible for the good wound‐healing results obtained after L‐PRF application. Furthermore, differences found over time, including up‐regulation of MMP9, TSP1, and CO3 and down‐regulation of fibrinogen and CATS at day 3, show the reactions that are taking place in the bio‐membrane at each moment and contribute to understanding the L‐PRF biological properties.

## 6 ANTI‐INFLAMMATORY CAPACITY

The presence of leukocytes in platelet concentrates is of great importance. Apart from providing antibacterial capacity, leukocytes play a role as immune regulator via the secretion of key immune cytokines such as IL‐1β, IL‐6, IL‐4, and TNF‐α. For instance, neutrophils are recruited to the site of injury within minutes following trauma and are the hallmark of acute inflammation. They migrate toward the damaged site and become embedded in the fibrin network to form a dense barrier against pathogens and prevent infection.

A recent systematic review of in vitro studies summarized in detail the anti‐inflammatory effect of L‐PRF matrices.^72^ For example, L‐PRF reduced the lipopolysaccharide (LPS)‐induced proinflammatory cytokine release murine ST2. In addition, Nasirzade et al.^40^ showed that L‐PRF reduced the expression of the M1 marker genes IL‐1β and IL‐6 in bone marrow macrophages. This anti‐inflammatory effect might be explained by the high amount of TGF‐β in L‐PRF capable of modulating the M2 and their polarization along with the generation of pro‐resolving lipid mediators. Following this research line, lipids from the solid and liquid forms of L‐PRF appeared to substantially decrease cytokine‐induced expression of IL‐6, CCL2, and CCL5 in murine ST2 cells. Moreover, L‐PRF lipids further reduced the LPS‐induced expression of IL‐1β, IL‐6, and CCL5 in macrophages at the transcriptional level. This was confirmed by showing the ability of PRF lipids to diminish IL‐6 at the protein level in ST2 cells and macrophages. These findings suggested that the lipid fraction of L‐PRF is at least partially responsible for the anti‐inflammatory activity of L‐PRF in vitro.^73^ Table 2 shows the most relevant studies regarding the anti‐inflammatory capacity of L‐PRF matrices.

**TABLE 2:** Most relevant studies evaluate the anti‐inflammatory effect of the L‐PRF matrices.

Study (year)	Cell type	L‐PRF preparation	Method	Main outcome
Mudalal et al. (2019)^177^	Human gingival fibroblast	3000 rpm/1 min Eppendorf Centrifuge 5702, Hamburg, Germany	Gene expression of IL‐1β, IL‐6, and TNF‐α by RT‐PCR	Decrease expression of IL‐1β, IL‐6, and TNF‐α
Nasirzade et al. (2020)^40^	Murine primary macrophages	1570 rpm/12 min Z306 Hermle Universal Centrifuge, Wehingen, Germany	Gene expression of M1 markers genes IL‐1β and IL‐6; M2 genes Arg1, Ym1a, lipoxygenases, ALOX12, and ALOX15 by RT‐PRC IL‐6 by ELISA; and NF‐B intracellular translocation	Decreased expression of IL‐1β and IL‐6 Decreased expression of Arg1, Ym1, and lipoxygenases Reduced IL‐6 protein level Reduced intracellular translocation of NF‐B
Kargarpour et al. (2021)^178^	Murine bone marrow‐derived ST2 cells	2000 g/8 min Z306 Hermle Universal Centrifuge, Wehingen, Germany	Gene expression of marker genes of IL‐1β, IL‐6, TNα, iNOS, and CLL5 in murine ST2, 3T3‐L1, calvaria cells, human gingival fibroblast, and HSC2	Decreased expression of IL‐6 and iNOS in pre‐adipocyte mesenchymal cells Not effective in reducing inflammatory response of human gingival fibroblasts
Kargarpour et al. (2021)^41^	Murine bone marrow‐derived macrophages and RAW 264.7 cells	2000 g/8 min Z306 Hermle Universal Centrifuge, Wehingen, Germany	Gene expression of IL‐6 by RT‐qPCR and secretion of IL‐6 by means of ELISA	Suppressed LPS‐induced inflammatory effect in RAW 264.7 cells M1‐to‐M2 polarization shift (from pro‐inflammatory M1 to pro‐resolving M2 phenotype) in murine bone marrow cells
Kargarpour et al. (2022)^4^	Murine RAW 234.7 macrophage cells	2000 g/8 min Z306 Hermle Universal Centrifuge, Wehingen, Germany	Gene expression of IL‐6 and iNOS COX‐2 by RT‐qPCR and secretion of IL‐6 by means of ELISA	Decreased expression of LPS‐induced IL‐6, iNOS, and COX‐2 Decreased inflammation induced by poly (1:C) HMW and FSL‐1 (agonist of Toll‐like receptor 3)

Abbreviations: ELISA, enzyme‐linked immunosorbent assay; MA2, macrophages 2; RT‐PCR, real‐time PCR.

**FIGURE 4:**
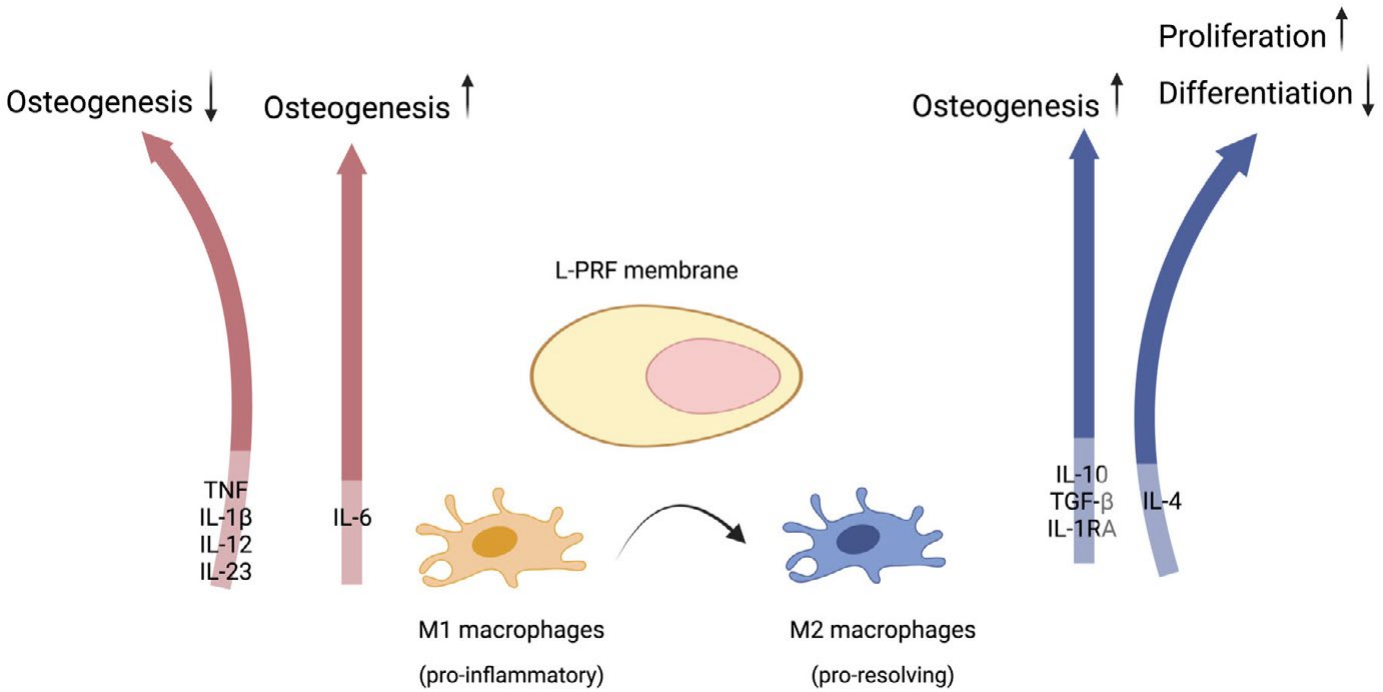
Schematic illustration of the shift from M1 to M2 promoted by the leukocyte and platelet rich fibrin membrane and the effects of the cytokines secreted on osteogenesis.

Macrophages have also been implicated in inflammatory processes. However, they also play an essential role in bone repair.^74^ During bone injury, monocytes and macrophages modulate the acute inflammatory response, produce growth factors such as bone morphogenetic protein‐2 (BMP‐2) and PDGF‐BB, and induce osteogenesis in mesenchymal stem cells.^75^, ^76^ Recently, it has been shown^41^ that L‐PRF could promote the M1‐to‐M2 polarization shift in murine bone marrow cells, which means a change from pro‐inflammatory M1 to pro‐resolving M2 phenotype (Figure 4). Similarly, Zhang et al.^77^ investigated the anti‐inflammatory effect of injectable PRF (i‐PRF) on immune response‐related cells, especially macrophages and dendritic cells. i‐PRF reduced pro‐inflammatory M1 phenotype of macrophages and inhibited the classical inflammatory‐related NF‐κB signal pathway. These results indicated the potential anti‐inflammatory role of i‐PRF during regeneration.

## 7 ANALGESIC CAPACITY

The use of fibrin adhesives has been documented as physiological additives that can modulate inflammation and increase the therapeutic effect postoperatively.^78^, ^79^ However, due to the risk of cross‐infection and different protocols for preparation, the use of these additives has been controversial, and thus limited. L‐PRF is characterized by a slow polymerization during preparation, which produces a fibrin network similar to that of the natural cells, which enhances cell migration and proliferation. As a reservoir of platelets, cytokines, leukocytes, and immune cells, L‐PRF allows a sustained release of cytokines such as VEGF, PDGF, TGF, and EGF that play a key role in vascular and tissue healing and scarring.^63^, ^80^, ^81^ Delayed wound healing causes problems for many patients both physically and psychologically, contributing to pain, economic burden, and loss of function.^82^ Therefore, L‐PRF may be an optimal adjunctive given it enhances angiogenesis and increases the coverage of the injured tissue by improving the proliferation of epithelial cells and fibroblasts, and thus reduces postoperative discomfort.^83^, ^84^

### 7.1 After tooth extraction

Tooth extraction is one of the most common procedures in Dentistry. However, in some cases, postoperative discomfort (i.e., pain and swelling) may drastically hinder patient's daily life for a few days. The use of L‐PRF has been presented as alternative to reduce this discomfort.^85^, ^86^ The highest expression of growth factors and cytokines in L‐PRF membranes occurs within the first 24 h,^62^ so this may be important in postextraction sockets for enhancing angiogenesis and accelerating soft tissue healing.

A recent systematic review and meta‐analysis assessed the effect of L‐PRF on postoperative discomfort after surgical extraction of the impacted mandibular third molars.^87^ Statistically significantly less postoperative pain and swelling was reported,^88^, ^89^, ^90^ as well as a reduced incidence of alveolar osteitis.^91^, ^92^ However, no statistically significant difference was observed between control group (spontaneous healing) and L‐PRF group with respect to trismus, osteoblastic activity, and soft tissue healing.^88^, ^93^, ^94^ Controversial results can be found in literature regarding postoperative pain after tooth extraction (no third molars) with the use of L‐PRF matrices. Statistically significant less pain and less analgesic consumption have been reported by de Almeida Barros Mourão et al.^95^ after 1 week. However, Ustaoğlu et al.^96^ showed less pain on day 1, but those differences were not significant after day 2. Similar results were observed by Temmerman et al.^97^ and Marenzi and et al.^98^ with a significant decrease in pain in the early healing phase (Table 3).

**TABLE 3:** Most relevant studies evaluate the influence of L‐PRF matrices on postoperative pain.

Study (year)	Number, gender, and age range	Control group	Test group	Methodology	Outcome
Follow‐up					
*Tooth extraction*					
Marenzi et al. (2015)^98^ 4 days	*N* = 26 ♂: 9 ♀: 17 (mean 53 years)	Spontaneous healing	L‐PRF 2700 rpm/12 min^a^	VAS	Significantly less pain in test group (*p* < 0.0001)
Temmerman et al. (2016)^97^ 7 days	*N* = 22 ♂: 15 ♀: 7 (mean 54 years)	Spontaneous healing	L‐PRF 2700 rpm/12 min^a^	Dutch version of the McGill Pain Questionnaire	Significantly less pain in test group on days 3.4 and 5 (*p* < 0.05)
Asmael et al. (2018)^179^ 3 days	*N* = 20 ♂: 28 (mean 44.2 y)ears	Spontaneous healing	L‐PRF 3000 rpm/10min^b^	VAS	No significant differences between groups (*p* > 0.05)
Girish Kumar et al. (2018)^180^ 7 days	*N* = 48 ♂: 28 ♀: 29 (mean 35.4 years)	Spontaneous healing	L‐PRF –	VAS	No significant differences between groups
de Almeida Barros Mourão et al. (2020)^95^ 1 week	*N* = 32 ♂: 19 ♀: 13 19–57 (mean 37 years)	Spontaneous healing	L‐PRF 2700 rpm/12 min^a^	VAS and number of consumed analgesic tablets	Significantly less postop pain in test group (T 4.0 ± 1.15 vs. C 5.12 ± 1.08) Less analgesic consumption in test group (T 1.0 ± 1.15 vs. 1.75 ± 0.85)
Ustaoğlu et al. (2020)^96^ 3 days	*N* = 57 ♂: 28 ♀: 29 (mean 35.4 years)	Spontaneous healing	T1: L‐PRF 2700 rpm/12 min^a^ T2: T‐PRF 2800 rpm/12 min^d^	VAS and number of consumed analgesic tablets	Significantly less pain in both test groups on day 1 (*p* = 0.047) No significant differences from day 2 between groups (*p* = 0.054) No significant differences in intake of analgesics (*p* > 0.05)
*Protection donor site*					
Femminella et al. (2016)^101^ 4 weeks	*N* = 40 ♂: 15 ♀: 25 (mean 32 years)	Gelatin sponge	L‐PRF 3000 rpm/10 min^a^	VAS and number of consumed analgesic tablets	Significantly less pain in the test group and less analgesic consumption (*p* < 0.05)
Ustaoğlu et al. (2016)^181^ 21 days	*N* = 40 ♂/♀: not reported (mean not reported)	Spontaneous healing	T‐PRF 1800 rpm/12 min^d^	VAS and number of consumed analgesic tablets	No significant differences in postoperative pain between groups during 1st week postop (*p* > 0.05) No significant differences in intake of analgesics (*p* > 0.05)
Ozcan et al. (2017)^102^ 4 weeks	*N* = 125 ♂/♀: not reported (mean)	Spontaneous healing	L‐PRF –	VAS	Significantly less pain in the test group (*p* = 0.0001)
Bahammam (2018)^103^ 7 days	*N* = 24 ♂: 14 ♀: 10 (mean 28 years)	Spontaneous healing	L‐PRF 3000 rpm/10 min^d^	VAS, NRS, and VRS	Significantly less pain in the test group (*p* < 0.05)
Sharma et al. (2019)^106^ 4 weeks	*N* = 20 ♂: 5 ♀: 15 (mean)	Collagen dressing	L‐PRF 3000 rpm/10 rpm^d^	VAS	No significant differences in postoperative pain between groups (*p* > 0.05)
Patarapongsanti et al. (2019)^104^ 4 weeks	*N* = 18 ♂: 7 ♀: 11 (mean 60 years)	Oxidized regenerated cellulose	L‐PRF 2700 rpm/12 min^a^	VAS	Significantly less pain in the test group during the first postoperative week (*p* < 0.05)
Sousa et al. (2020)^105^ 3 months	*N* = 25 ♂: 9 ♀: 16 (mean 36 years)	Gelatin sponge	L‐PRF 1500 rpm/8 min^c^	VAS	Significantly less pain in the test group on day 2 (*p* = 0.013)
Kızıltoprak and Uslu (2020)^182^ 3 months	*N* = 36 ♂: 9 ♀: 27 18–53 (mean not reported)	Spontaneous healing	L‐PRF 2300 rpm/3 min^c^ Autogenous fibrin glue (AFG) 2700 rpm/2 min^c^	VAS	No significant differences in postoperative pain between groups at day 3 (*p* > 0.05) Significantly less pain for AFG compared to L‐PRF and control at day 7 (*p* < 0.05)
*Sinus floor elevation (lateral window approach)*					
Del Fabbro et al. (2013)^183^ 7 days	*N* = 30 ♂: 12 ♀: 18 37–66 (mean not reported)	DBBM	DBBM + PRGF	VAS	Significant reduction of pain compared to control group (*p* < 0.05) After day 4, similar results for the two groups
Gurler and Delilbasi (2016)^110^ 7 days	*N* = 28 (24) ♂: 14 ♀: 10 23–66 (mean 46.3 years)	Allogenous bone graft Collagen membrane covering window	Allogenous bone graft L‐PRF membranes covering window 2700 rpm/12 min^a^	VAS	No statistically significant differences between groups (*p* > 0.05)

Abbreviations: DBBM, deproteinized bovine bone mineral; NRS, numeric rating scale; VAS, visual analog scale; VRS, verbal rating scale.

^a^ IntraSpin centrifuge, BioHorizons, Birmingham, USA.

^b^ Xiangtian, Jiansu, China.

^c^ DUO‐Quattro, Process, Nice, France.

^d^ Not mentioned.

### 7.2 Management of palatal wounds after graft harvesting

Many tissue‐harvesting procedures have been described during mucogingival surgery. The most commonly used technique, the free gingiva graft (FGG), is easy to perform and enables the harvest of large quantities of high‐quality connective tissue. Conversely, it produces a site of secondary‐intention wound healing that may create discomfort and pain.^99^ L‐PRF has been recommended for use as a palatal bandage to cover the donor site after FGG harvesting as it enhances cell migration and proliferation, and thus cicatrization.^84^, ^100^

According to literature, significantly less pain was often reported when L‐PRF membranes were used to protect the palate donor site.^101^, ^102^, ^103^, ^104^, ^105^ This was accompanied by less intake of analgesics during the early healing phase. Two of eight studies reporting on the use of L‐PRF for the management of palatal wounds could not find statistically significant differences between spontaneous healing^96^ or a collagen dressing^106^ compared to L‐PRF membranes.

### 7.3 Postoperative pain after sinus floor elevation

Postoperative pain, swelling, and edema are the most frequent complications after sinus lifting.^107^ Evidence of the effect of L‐PRF on postoperative discomfort after sinus lift is scarce. There are no studies evaluating the postoperative complications after the use of L‐PRF as a sole‐filling material. Recently, Chen et al.^108^ reported significantly more discomfort when a bone grafting material (deproteinized bovine bone mineral) was used in combination with L‐PRF during osteotome sinus floor elevation. Regarding the two‐stage lateral window approach, Del Fabbro et al.^109^ reported significantly less pain when PRGF was added to the bone graft (deproteinized bovine bone mineral) (*p* < 0.05). However, similar results for the two groups were observed after day 4 postop. Likewise, Gurler et al.^110^ could not find any difference in pain relief after the use of L‐PRF membrane to cover the lateral window compared to a collagen membrane.

## 8 ANTIBACTERIAL CAPACITY

Antimicrobial activity can be defined as the destruction or inhibition of the growth of microorganisms (bacteria, fungi, and viruses).^111^ In the last decade, numerous studies have been performed to determine and characterize the antimicrobial activity of L‐PRF. Recently, a systematic review of in vitro studies concluded that all types of PRF showed significant antimicrobial action, with the antibacterial efficacy being more expressive than the anti‐fungal one.^112^ The exact components/pathways responsible for the antimicrobial activity in platelet concentrates (PCs) have not been clearly established yet, but there are several theories on which mechanisms and conditions are involved according to the PCs' content.^43^

L‐PRF is characterized by an increased concentration of platelets, which are considered cells with a multifunctional role in antimicrobial host defense.^113^ Numerous peptides with antimicrobial properties, such as platelet factor 4 (PF‐4), connective tissue‐activating peptide 3 (CTAP‐3), thymosin beta 4 (Tβ‐4), platelet basic protein (PBP), and fibrinopeptide B (FP‐B), and oxygen metabolites such as superoxide, hydrogen peroxide, and hydroxyl free radicals, are released by platelets after being stimulated by microorganisms.^113^

Furthermore, L‐PRF contains a large number of already well‐known important immune cells: the leukocytes. After its activation, neutrophils migrate to the site of infection, pathogens are exposed to released antimicrobial substances either in the phagosome or extracellularly. Neutrophils contain primary and secondary granules with enclosed antimicrobial proteins, peptides, and enzymes, such as lactoferrin, defensins, bactericidal/permeability‐increasing protein (BPI), azurocidin‐/heparin‐binding protein, cathelicidins, phospholipases A2, and calprotectin. Another cell type that reaches an even higher concentration than other leukocytes are lymphocytes, which are involved in innate and adaptive immune responses.^114^, ^115^ Lymphocytes are activated by bacterial products or cytokines and carry out immune responses either through mediators or directly.^20^, ^115^ Another system that may provide antimicrobial activity in the use of PCs is the complement system, which activates the complement cascade and promotes the lysis of microorganisms. The complement system also stimulates leukocyte recruitment for humoral defense realization.^116^

Several studies have evaluated the antibacterial effect of L‐PRF (Table 4). Overall, L‐PRF showed bacterial growth inhibition against the main periodontal pathogens (*A. actinomycetemcomitans*, *P. gingivalis*, *P. intermedia*, and *F. nucleatum*).^42^, ^43^, ^44^, ^45^ However, preparation protocols differed enormously among studies and may hinder the real comparison between them. For instance, Yang et al.^42^ prepared L‐PRF by adding calcium chloride to PRP in order to activate the platelets and convert fibrinogen into fibrin, which is not the standard protocol for preparing L‐PRF. Therefore, these results must be interpreted with caution.

**TABLE 4:** Most relevant studies evaluating the antimicrobial effect of L‐PRF matrices.

Study (year)	Cell type	L‐PRF preparation	Methodology	Results
Yang et al. (2015)^42^	*Aggregatibacter actinomycetemcomitans* *Porphyromonas gingivalis* *Fusobacterium nucleatum*	PRP: 500 g, 10 min, and 6000 g/30 min L‐PRF: fraction of PRP activated with CaCl_2_, 6000 g/30 min	Microdilution test Time‐Kill assay + CFU counting on agar plates Adhesion assay	Bacteriostatic properties against all bacteria Bacterial inhibition against *P. gingivalis* up to 36 h Inhibition of adhesion against *A. actinomycetemcomitans* and *P. gingivalis*
Kour et al. (2018)^43^	*A. actinomycetemcomitans* *P. gingivalis*	i‐PRF: 700 rpm/3 min L‐PRF: 3000 rpm/10 min PRP: 1000 rpm/3 min and 2000 rpm/10 min	Agar diffusion test	Bacterial growth inhibition in all groups *P. gingivalis*: i‐PRF had the widest zone (*p* < 0.05) *A. actinomycetemcomitans*: PRP had the widest zone (*p* < 0.05)
Castro et al. (2019)^44^	*A. actinomycetemcomitans* *P. gingivalis* *Prevotella intermedia* *Fusobacterium nucleatum*	L‐PRF: 2700 rpm/12 min	Agar diffusion test Microdilution test	L‐PRF membrane: effect on all bacteria L‐PRF exudate showed strong effect on *P. gingivalis*, and decreased viability of *P. gingivalis* in a dose‐dependent way
Feng et al. (2020)^116^	*Staphylococcus aureus* *Escherichia coli*	L‐PRF: 700 g/12 min H‐PRF: 700 g/8 min	Agar diffusion test Disk diffusion test CFU counting on agar plates Flow cytometry	Both PRF matrices showed antibacterial effect (H‐PRF > L‐PRF) against both bacteria (*E. coli* > *S. aureus*)
Jasmine et al. (2020)^118^	*S. aureus* *Staphylococcus epidermidis*	i‐PRF: 1000 rpm/5 min	Microdilution test Live/dead assay Confocal laser scanning microscopy	MIC: 80 μL/mL in nonbiofilm bacteria MBC: 160 μL/mL in nonbiofilm bacteria Inhibition of biofilm formation after 24 h L/D assay: 50% dead cells at MIC and 100% dead cells at MBC
Rodríguez Sánchez et al. (2021)^117^	*P. gingivalis* Multispecies biofilm	L‐PRF: 2700 rpm/12 min	Agar diffusion test Microdilution test	*P. gingivalis* was inhibited by peroxidase or pepsin released by L‐PRF exudate L‐PRF exudate showed antimicrobial effect on developing multispecies biofilms No antibacterial effect on pre‐formed multispecies biofilm
Pham and Tran (2023)^45^	*A. actinomycetemcomitans*	A‐PRF+: 1300 rpm/8 min i‐PRF: 700 rpm/3 min	Microdilution test Time‐kill assay + CFU counting on agar plates	i‐PRF better antiadhesion effect than A‐PRF+ (*p* = 0.012) Bacterial growth reduction (80% after 12 h), by 48 h similar proliferation as control

Abbreviations: A. acetinomycetemcomitans, Aggregatibacter acetinomycetemcomitans; P. gingivalis, Porphyromonas gingivalis; P. intermedia, Prevotella intermedia; F. Nucleatum, Fusobacterium nucleatum, CFU, colony‐forming unit; S. aureus, Staphylococcus aureus; E. coli, Escherichia coli; S. Epidermis, Staphylococcus epidermis; MIC, minimum inhibitory concentration; MBC, minimum bactericidal concentration; L/D assay, live/dead assay.

The exact mechanisms underlying this antimicrobial effect have not been completely demonstrated yet. Rodríguez Sánchez et al.^117^ aimed to characterize those mechanisms involved in the antimicrobial effect of L‐PRF against *P. gingivalis*. The antimicrobial effect was blocked in all in vitro models by exposing the L‐PRF exudate to horseradish peroxidase. Pepsin showed similar blocking effect on L‐PRF exudate, with the exception of the developing multispecies model. The authors highlighted that the antibacterial effect might rely on the release of peroxidase and pepsin from the L‐PRF derivates. A preceding study found that L‐PRF contained immunoglobulin G (IgG) that may provide some infection control against periodontal bacteria during the postoperative wound‐healing period.^66^ Another study investigating injectable PRF (i‐PRF) reported that its antimicrobial and antibiofilm activity was probably related to permeability proteins.^118^ The authors of this study also stated that the inhibitory and bactericidal effect of i‐PRF was due to its composition of platelets, fibrin, fibronectin, thrombin, HBD‐3 peptide (antimicrobial peptide), myeloperoxidase, and inclusion of white blood cells.

Recently, the possibility of combining antibiotics with L‐PRF to increase the antibacterial effect has been explored. Polak et al.^119^ showed for the first time that incorporating antibiotics (metronidazole 5 mg/mL, clindamycin 150 mg/mL, or penicillin 1 mU/mL) to L‐PRF matrices increased the antibacterial effect against *F. nucleatum* and *Staphylococcus aureus*. Therefore, the modified L‐PRF membranes may be used as a locally sustained released device for antibiotics. Following this idea, Dubnika et al.^120^ and Ercan et al.^121^ incorporated antibiotics (vancomycin and doxycycline, respectively) in the L‐PRF matrices in vitro. Both reported higher antibacterial capacity and a long‐acting effect in test groups. When extrapolating these results to clinical practice, it is very interesting to find out if patients taking antibiotics before surgical treatment would incorporate them into the L‐PRF. Siawasch et al.^122^ evaluated the effect of administering systemic antibiotics (amoxicillin 2 g or metronidazole 500 mg) on the release of growth factors and the antibacterial activity of the L‐PRF membranes. The antibacterial capacity increased significantly after systemic intake, without disadvantage for the release of growth factors. Clinically, this might be taken into account when treating patients with high risk of medication‐related osteonecrosis of the jaws (MRONJ), ridge preservation after extraction of an infected tooth, or during periodontal regenerative surgery.

## 9 HEMOSTATIC EFFECT

Hemostasis is the mainstay of every successful surgery, playing a critical role when the surgery takes place in an extensive region. Over the last decades, the number of medically compromised patients being treated with continuous anticoagulant therapy has increased enormously. Therapy suspension reduces a potential hemorrhage risk, exposing patient to thromboembolic risk. Similarly, drug dosage maintenance determines a greater probability of intra‐ and postoperative bleeding, safeguarding the patient from the formation of potential clots. Current guidelines contemplate the possibility of not suspending or modifying anticoagulant therapy for minor surgical procedures, preventing bleeding complications by using hemostatic agents (fibrin glue, oxidized cellulose, and topical antifibrinolytics) in the surgical site.^123^, ^124^

The rationale for the use of L‐PRF as a hemostatic agent lies in its intrinsic property of facilitating blood clot formation through rapid activation of the coagulation cascade. Furthermore, the formation of a physical barrier protecting the surgical site should not be underestimated.^125^, ^126^ Thanks to its three‐dimensional architecture, L‐PRF membranes offer greater biomechanical characteristics than other platelet concentrates.^127^ This property facilitates membrane adaptation to different surgical sites, allowing its stabilization on soft tissues by compression or suturing.

### 9.1 Anticoagulant medication

Regarding patients taking vitamin K antagonists, Sammartino et al.^125^ explored the effectiveness of L‐PRF membranes in the prevention of hemorrhagic events after tooth extraction without modifying the anticoagulant therapy. Only 2 of the 50 patients (4%) reported a hemorrhagic complication, which could be solved by compression and hemostatic topical agents. Twenty percent of the included patients presented mild bleeding and the remaining 76% showed an adequate hemostasia. More recently, Berton et al.^124^ performed a cohort observational study to assess the efficacy of L‐PRF plug as hemostatic agent in single tooth extraction in patients under treatment with vitamin K antagonist (VKA: warfarin or acenocumarol) or direct oral anticoagulant (DOACs: dabigatran, rivaroxaban, apixaban, or edoxaban). Postoperative bleeding was recorded in 9 of 53 patients (17%) with VKA and 9 of 59 patients (15.3%) for DOACs group. None of the patients needed medical support for managing bleeding.

Direct thrombin inhibitors or factor Xa inhibitors (DOACs) have been extensively studied and showed a more predictable and less labile anticoagulant effect compared to standard anticoagulant therapy. de Almeida Barros Mourão et al.^128^ followed 25 patients on anticoagulant therapy with factor Xa inhibitor (rivaroxaban or apixaban) requiring single tooth extractions. In all patients, L‐PRF clots were packed into the extraction socket and stabilized with an overlying cross suture. The L‐PRF clots successfully arrested bleeding in all patients and no complications were observed at any time point.

### 9.2 Antiplatelets medication

The biology of platelet activation and aggregation is becoming better understood and has provided numerous molecular targets for drugs to establish an antiplatelet effect. Giudice et al.^129^ compared four different interventions after tooth extraction in patients taking oral antiplatelet medication in a split‐mouth randomized clinical trial: (1) suturing alone, (2) suturing + hemostatic plug, (3) suturing + A‐PRF+ plug, and (4) suturing + L‐PRF plug. Both L‐PRF plugs resulted in less postextraction bleeding (1/40 and 2/40 patients, respectively) compared to the hemostatic plug (5/40) or the suturing alone (8/40). Statistically significant less bleeding could be observed for A‐PRF+ when compared to suturing alone (*p* < 0.03).

## 10 DIFFERENCE BETWEEN L‐PRF MODIFICATIONS

Recently, new L‐PRF modifications have been proposed reducing the relative centrifugal force and duration of the centrifugation.^130^, ^131^ By reducing the relative centrifugal force (the low‐speed centrifugation concept), an increase in the release of growth factors and in the concentration of leukocytes and platelets was envisaged. Theoretically, less centrifugation time would reduce pull‐down forces during centrifugation, which would increase the total number of cells contained within the L‐PRF clot. Ghanaati et al.^131^ reported a higher concentration of cells at the red part of the L‐PRF clot (face, part in contact with the red blood cells) compared to A‐PRF (advanced platelet‐rich fibrin) where the cells were distributed throughout the clot. The same group of researchers formulated another PRF preparation called A‐PRF+ (advanced platelet‐rich fibrin), reducing even more the g‐force and the duration of centrifugation (207 g, 8 min). A significantly higher release of growth factors after 10 days as well as greater migration of human gingival fibroblasts to the A‐PRF and A‐PRF+ was reported compared to L‐PRF. In 2019 Miron et al.^132^ introduced a new protocol with horizontal centrifugation, based on other medical centrifuges that utilize horizontal swing‐out bucket rotors to separate layers based on density. They also reported an even higher concentration of cells compared to L‐PRF and A‐PRF with an even distribution of cell types throughout the fibrin matrix.

When comparing these last L‐PRF modifications, qualitative morphological analysis revealed that whole platelets were distributed widely and homogeneously in H‐PRF and A‐PRF, but localized along the distal tube surface in L‐PRF. Activated platelets were distributed as were whole platelets in A‐PRF and L‐PRF, but localized mainly in the “buffy coat” region in H‐PRF. No significant difference in the ratio of activated to whole platelets between PRF derivatives could be detected. These findings suggest that platelet activation is similarly induced in fibrin matrices regardless of centrifugal speed or rotor angulation. However, only the H‐PRF group was distinguishable from the other PRF derivatives in terms of activated platelet distribution.^133^

In vitro studies comparing A‐PRF or A‐PRF+ with L‐PRF have led to controversial data.^62^, ^134^, ^135^ For instance, Dohan Ehrenfest et al.^136^ compared L‐PRF versus A‐PRF prepared with various centrifugation devices and concluded that the L‐PRF protocol allowed the production of larger clots/membranes and a more intense release of growth factors. In contrast, in a similar study, El Bagdadi et al.^130^ compared L‐PRF versus A‐PRF versus A‐PRF+ and observed an increase in growth factors release when RCF was reduced. Comparing findings is complicated by the heterogeneity in methods used, such as type of tube (plastic or glass) and adaptation of RCF to the rcf‐max or rcf‐clot. Moreover, neither of these studies evaluated the real effect of the centrifuge when the same PRF matrices were prepared with the g force adapted for each device nor the impact of using a glass or plastic tube. Moreover, the clinical relevance of the difference centrifugation protocols still needs to be demonstrated in clinical studies.

The development of the liquid PRF formulations allowed the easy combination with bone substitutes to create stronger constructs. Among injectable platelet concentrates, platelet‐rich plasma (PRP) was the first PC used in regenerative medicine.^137^, ^138^ Nevertheless, the preparation of PRP typically requires two centrifugation steps and uses anticoagulants added to the blood collection tubes. As a result, injectable platelet‐rich fibrin (i‐PRF), a second‐generation platelet concentrate, was developed in 2015 by modifying the relative centrifugal force (RCF), time of centrifugation, and by using more hydrophobic plastic blood collection tubes.^139^ This liquid PRF formulation was originally prepared using a single centrifugation cycle (60 g, 3 min) without the need/addition of any anticoagulants^140^ and demonstrated the ability to release higher concentrations of various growth factors, and induced higher fibroblast migration and expression of PDGF and TGF‐β than PRP.^141^ By using lower centrifugation speeds, shorter centrifugation time, and hydrophobic plastic tubes, the fibrin clotting times could be drastically delayed, leaving a final liquid PRF formulation following the centrifugation cycle. The resulting i‐PRF was shown to contain thrombin and fibrinogen, thus making it suitable to be injected for periodontal injection^142^ or mixed with bone substitutes.^143^

Cortellini et al.^144^ described a new guided bone regeneration technique with the so‐called L‐PRF bone block, also known by many clinicians as “sticky bone”.^145^ This block was prepared by mixing particulate biomaterial (deproteinized bovine bone mineral, DBBM) with chopped L‐PRF membranes and adding liquid fibrinogen to keep all the components together, and has been used for horizontal bone regeneration^144^ or in sinus floor elevations.^146^ The liquid fibrinogen was prepared with the same *g* force as for L‐PRF (408 g at the rcf‐clot) but by reducing the time (3 min) and presented a high concentration of cells (88% platelets, 72% leukocytes).^63^ This liquid form of L‐PRF has shown to release growth factors overtime which appeared to be correlated with platelet concentration.^147^ Moreover, several in vitro studies concluded that DBBM can support osteoblast attachment and proliferation, and can be further enhanced when PDGF is on the surface.^148^, ^149^ This would support the hypothesis that growth factors on/or near the xenograft can influence osteogenesis in vivo. Likewise, Schwartz et al.^150^ suggested that DBBM might have tissue‐specific osteoinductive properties and might contain growth factors such as TGF‐β1 and BMP‐2. Given the active production of growth factors by the L‐PRF membrane and derivatives, it might further stimulate the biological properties of the DBBM particles becoming a bioactive scaffold when bone regeneration needs to be achieved.

## 11 EFFECT OF L‐PRF ON SOFT TISSUE REGENERATION

### 11.1 Biological mechanisms

Wound healing remains a challenging clinical problem and correct and efficient wound management is essential. Despite the fact that the processes of repair begin immediately after an injury in all tissues and that all wounds go through similar phases, there are differences between tissues in terms of the time required to complete regeneration.^151^, ^152^ Recently, it has been envisaged that L‐PRF may accelerate wound healing^153^ by stimulating fibroblast wound closure in vitro^84^, ^154^ and promoting the ability of fibroblasts to induce endothelial tube formation.^83^ It has also been found that L‐PRF was able to increase cell proliferation in a number of cells implicated in soft tissue repair, induce the mitogenic activity of endothelial cells important for angiogenesis, and release an array of growth factors to the surrounding microenvironment.^155^ These growth factors are chemotactic for various cell types, including monocytes, fibroblasts, endothelial cells, stem cells, and fibroblasts, creating tissue microenvironments and directly influencing the proliferation and differentiation of progenitor cells.^156^

### 11.2 Current evidence

The effects of L‐PRF on soft tissue wound healing and angiogenesis have been investigated in various animal models. Roy et al.^157^ reported significantly improved angiogenesis in chronic wounds and collagen matrix deposition in a porcine ischemic excision wound model after 14 days for the L‐PRF group compared to control (unassisted healing). In another subcutaneous implantation model performed in mice, L‐PRF readily integrated with surrounding tissues, and 2 weeks after implantation, it was partially replaced by collagen fibers.^158^ In a model designed to regenerate the parotid gland after their irradiation in minipigs, both adipose‐derived stem cells and L‐PRF significantly sped the repair of defects in maxillofacial soft tissue in irradiated minipigs, and their combined use was more effective after a 6‐month healing period.^159^ In 2014, it was found that L‐PRF increased type 1 collagen formation in full‐ and split‐thickness flaps and improved skin graft take in a skin graft model performed in porcine animals.^160^ Thus, these studies showed that L‐PRF is able to increase soft tissue wound healing in various animal models, and reports document that this is primarily due to the increase in angiogenesis to defect sites.

Clinically, L‐PRF has been used as a protection of the resorbable barrier membranes in GBR procedures, in case a flap dehiscence after bone augmentation occurs.^144^ Talon et al.^161^ reported a higher cell adhesion and spreading on the expanded polytetrafluoroethylene (e‐PTFE) membranes when coated with L‐PRF. This is of relevant importance with this kind of membrane because of the high ratio of flap dehiscence or flap perforation.^162^ The same principle is applied when L‐PRF is used at the palatal donor site after harvesting a free gingival graft or a connective tissue graft. Various studies have shown faster wound healing and less postoperative discomfort when applying L‐PRF in wound.^101^, ^163^

## 12 EFFECT OF L‐PRF IN HARD TISSUE REGENERATION

### 12.1 Biological mechanisms

Aside from its effect on soft tissues, the application of L‐PRF has also been associated with positive results in bone tissue repair and regeneration. Bone graft healing starts with a blood clot formation primarily mediated by fibrinogen and fibrin polymerization. This process leads to a wide range of biological effects like growth factor binding and cellular interaction among platelets, fibroblasts, leukocytes, endothelial cells, and circulating stem cells. Thus, the rationale for the biopolymer fibrin as a scaffold for bone tissue engineering is an obvious possibility. Fibrin has been demonstrated to be a suitable scaffold material for colonization of human mesenchymal stem cells (HMSC),^164^, ^165^ and that they could adhere, spread, and proliferate, depending on different fibrinogen concentrations.^166^ The most commonly used fibrin scaffolds in tissue engineering are fibrin microbeads, fibrin glue, and fibrin hydrogels.^167^ Recent publications revealed the possible detrimental effect of the inclusion of leukocytes within platelet‐rich derived scaffolds.^32^, ^168^

L‐PRF is by definition an autologous fibrin matrix in which cells, growth factors, and cytokines are trapped and delivered over time.^127^ These cytokines stimulate the mitogenic response of the periosteum during early stage of bone repair^169^ and are clearly associated with bone‐healing processes.^170^ Several growth factors have been identified in L‐PRF affecting bone repair such as TGF‐β, FGF, VEGF, IGF, PDGF‐AB, and PDGF‐BB. For instance, PDGF is well known as an important regulator for the migration and proliferation of mesenchymal cells. Moreover, VEGF growth factor induces the angiogenesis process, and as a result, it improves the blood supply of the damaged tissue.^171^ It has been shown that TGF‐β in particular stimulates the synthesis of collagen and fibronectin in a variety of cell lines^172^ and that it increases the expression of integrins that bind collagen, fibronectin, and vitronectin.^173^ In this context, Park et al.^174^ showed that fibrin scaffolds containing cytokines like FGF and osteoblasts lead to higher bone formation than fibrin‐containing osteoblasts alone. Thus, this highlighted the importance of the presence of growth factors for new bone formation.

### 12.2 Current evidence

It is well established that osteoblasts are critical for the synthesis and mineralization of extracellular bone matrix. In this sense, Gassling et al.^175^ compared a commonly used porcine collagen membrane with L‐PRF membranes as scaffolds for periosteal tissue engineering. L‐PRF appeared to render superior results in terms of the proliferation of human osteoblasts and periosteal cells in vitro. In a similar study, the same group evaluated the capacity of L‐PRF membranes to serve as a scaffold for human osteoblast cells compared to the same porcine collagen membrane.^176^ Both membranes appeared to be suitable as scaffold for human osteoblast cells, however, L‐PRF membranes demonstrated superior cell proliferation and significantly higher alkaline phosphatase activity.

## 13 CONCLUSION AND FUTURE PERSPECTIVES

The use of L‐PRF matrices started already more than 20 years ago, but we are still trying to understand its impact on wound healing. Above all, there is a lack of understanding of its molecular and cellular mechanisms, especially regarding the extent to which we can exogenously stimulate an organism. When growth factors are incorporated into a bone graft, concentrations can often be 1000–100 000 times higher than what is naturally released by the L‐PRF. Therefore, further research needs to focus on determining the saturation threshold and discard any possible adverse effect of this excessive stimulation.

There is quite strong evidence that L‐PRF matrices enhance soft tissue healing. However, their application for bone regeneration somehow remains controversial. Even though it has been demonstrated that L‐PRF stimulates the migration and proliferation of osteoblasts, the clinical results seem to be operator sensitive. As for any other surgical technique or biomaterial, the correct handling is essential. Well‐designed studies with strict and rigorous protocols are necessary to accurately assess the true effect of L‐PRF in bone regeneration.

Another crucial perspective to be evaluated is how we can further improve the characteristics of L‐PRF to maximize its advantages. For instance, by using L‐PRF as carrier in drug delivery system, containing antibiotics or antimicrobials. Nevertheless, one needs to be aware that variations in blood composition among patients can influence the properties of L‐PRF. Patient's characteristics need to be taken into account when using platelet concentrates. Thus, a correct patient selection should be mandatory to assure predictable outcomes. However, the impact of systemic health conditions, such as diabetes, anticoagulant medication use, and (auto)‐immune diseases, on the L‐PRF composition and function still needs to be further studied.

The understanding of the benefits and limits of L‐PRF seems of utmost importance to avoid controversial results and to provide the best treatment to our patients.

## CONFLICT OF INTEREST STATEMENT

All (co)‐authors declare that they have no conflict of interest. The Department of Oral Health Sciences, Unit of Periodontology at KU Leuven owns research chairs from several implant companies: Dentsply Sirona, Straumann, and Henry Schein.

## Data Availability

Data sharing is not applicable to this article, as no new data were created or analyzed in this study.
